# Effects of Chicken Egg Powder, Bovine Colostrum, and Combination Therapy for the Treatment of Gastrointestinal Disorders

**DOI:** 10.3390/nu16213684

**Published:** 2024-10-29

**Authors:** Raymond John Playford

**Affiliations:** School of Medical & Biomedical Sciences, University of West London, St Mary’s Road, Ealing, London W5 3TX, UK; ray.playford@uwl.ac.uk; Tel.: +44-20-8231-2468

**Keywords:** nutraceuticals, bioactives, bioactivity, functional foods, growth factors, immunity, gut repair, gastrointestinal disease, inflammation

## Abstract

Natural-based products are of interest to the pharmaceutical industry as potential sources of novel medicinal compounds. They are also used by consumers/patients as standalone therapies or as an adjunct to Western medicines. Two natural-based products of interest are chicken egg and bovine colostrum (the milk produced in the first few days following calving). Both products are rich in immunoglobulins, antimicrobial peptides, growth factors, and macro- and micro-nutrients. In vitro, in vivo, and a limited number of clinical studies suggest therapeutic benefits of both components given alone and together. Combination therapy is of particular interest, as preclinical studies suggest synergistic effects on growth, repair, and gut protection, including microbiome-induced damage. This article describes the main constituents of egg and bovine colostrum, studies of their use alone and together for a wide range of conditions, highlights areas requiring further research, and describes novel indications such as GLP-1-associated gut symptoms. While well placed in the food supplement arena, additional high-quality clinical trials are required to establish their benefits in clinical practice.

## 1. Introduction

Medicinal products derived from nature have been used for thousands of years for a wide range of ailments [1,2] and are part of a global multi-million-dollar industry [3]. Although many products have been replaced by a single compound “pharmacological medicine”, they still offer potential to support overall health. Pharmaceutical companies routinely screen materials, such as plants, for sources of novel medicinal compounds. Often, patients take natural-based products in addition to Western medicines, in the hope of enhancing their efficacy or reducing their side effects [4,5]. However, the major market in natural-based products has the intension of preventing, as opposed to treating, disease and is often used for prolonged periods. Product safety is, therefore, of particular importance [6,7,8].

Natural products with pharmaceutical activity are sometimes termed *nutraceuticals*, derived from combining the term nutrition with pharmaceuticals. Two nutraceutical products that have potential value for the treatment of a wide range of conditions, including gastroenterological, are chicken egg and bovine colostrum (BC), used individually or together. These products distinguish themselves for particular attention as they are both natural-based, originate from normal food products, and have *GRAS* (generally regarded as safe) status in the USA [9,10,11]. In addition, in contrast with many other products marketed by the health supplement industry, there is a strong preclinical science publication base demonstrating their effects in a wide range of in vitro and in vivo situations, which is supported by a limited number of clinical trials. This review provides (1) an overview of the constituents of chicken egg and bovine colostrum, (2) a description of studies of their use alone and together for a wide range of conditions, including evidence for synergistic activity of the combination, (3) highlights areas for further research, and (4) describes the limitations of studies performed to date.

## 2. Constituents of Chicken Egg Powder

Eggs are considered nutrient-rich foods containing high levels of macro- and micro-nutrients [12,13,14]. These major components are discussed below. For a more detailed review of individual chemical constituents, see the review published by Réhault-Godbert et al. [12].

### 2.1. Macro-Nutrients and Micro-Nutrients

#### 2.1.1. Proteins

Egg proteins are equally distributed between egg yolk and white, constituting about 13% of total egg weight, along with 10% fat but with minimal carbohydrate (<1%) [12]. Hundreds of different proteins have been identified within an egg, the most abundant being ovalbumin, accounting for about 54% of total protein, with the remaining major constituents comprising ovotransferrin (12%), ovomucoid (11%), ovoglobulin (8%), ovomucin (3.5%), and lysozyme (3%) [15]. Within the yolk, albumin, ovalbumin, immunoglobulins apolipoprotein B, apovitellenin-1, vitellogenin, and ovotransferrin are the most abundant proteins [12,15].

#### 2.1.2. Fats and Lipids

Total lipid content is approximately 10 g per 100 g of the whole egg and is mainly concentrated in the egg yolk [12]. The yolk is rich in essential fatty acids such as linoleic acid (FA 18:2 9c,12c (*n*-6) and is relatively high in cholesterol (400 mg per 100 g of the whole egg) [12,16,17].

#### 2.1.3. Vitamins and Minerals

Egg is a rich source of vitamins B_12_, D, riboflavin, and choline; minerals such as phosphorus, calcium, and potassium; and contains all essential trace elements such as copper, selenium, zinc, iron, magnesium, and manganese [12].

### 2.2. Bioactive Components

In addition to macro- and micro-nutrients, egg is a rich source of compounds, particularly proteins/peptides, that may influence physiological processes such as growth, repair, and immunity [12]. Importantly, the presence of bioactive compounds in any orally ingested product does not equate to a definite ability to be absorbed and reach its target receptors intact (see the limitations section discussed later). Some of the major bioactive components of egg are briefly discussed below and are grouped according to their most well-established effects.

#### 2.2.1. Antimicrobial Factors

Probably the most studied antimicrobial factor within eggs is the immunoglobulin (Ig) component, the main form being IgY [18,19,20,21]. This has a functional resemblance to mammalian IgG, with two heavy chains and two light chains. It differs from IgG by not combining with human Fc receptors and does not possess a hinge region [19,20]. The relative advantage of IgY versus IgG is dependent on the clinical situation, but some potential advantages of IgY over IgG include greater specificity due to the lack of hinge region and the ability to neutralize pathogens without inducing an excessive inflammatory response, due to not binding complement. Readers interested in more detail are referred to the article by León-Núñez et al. [20].

In addition to IgY, several other components of egg exhibit antimicrobial activity against bacteria, parasites, and fungi [21,22]. Some peptides/proteins act through permeabilizing bacterial cell walls, e.g., lysozyme and avian beta defensins, while others act through decreasing the bioavailability of iron (ovotransferrin), vitamins (avidin), or through the inhibition of bacterial proteases (ovoinhibitor, cystatin) [21,22]. The proteolytic digestion of ovalbumin by trypsin or chymotrypsin has also been shown to produce peptide fragments with bactericidal activity [23].

#### 2.2.2. Cytokines and Antioxidants

Cytokines are peptides or proteins that influence immune activation, cell signaling, and recognition of pathogens. Egg contains many proteins, which, along with their partial hydrolysates, can influence immune responses when tested in in vitro models of inflammation. These include lysozyme, sulfated glycopeptides generated by proteolysis from ovomucin and chalazae, and pleiotrophin. For further details, see the review by Réhault-Godbert et al. [12]. Several egg proteins also possess antioxidant activity, with some of the same proteins possessing both cytokine and antioxidant activity. Proteins with antioxidant activity include ovotransferrin, ovomucoid, ovomucin, and egg yolk proteins, including phosvitin [12,24]. The clinical relevance of these proteins when ingested by humans in the form of egg is unclear, as there are only limited bioavailability studies and minimal clinical data. Further research is, therefore, required regarding their clinical relevance, efficacy, and safety [24].

#### 2.2.3. Growth Factors

Both raw and pasteurized egg powders exhibit pro-proliferative and pro-migratory bioactivity against a variety of cell lines originating from the stomach and small and large intestine [25]. Studies regarding which factors are most relevant in inducing these effects are less progressed than those with BC. Pro-proliferative and migratory activity against human gastrointestinal cell lines has been found in both yolk and egg white components, with ovomucoid and ovalbumin being the major likely contributors [25,26]. Interestingly, much of the pro-proliferative activity could be prevented by the co-presence of the epidermal growth factor (EGF) receptor blocker tyrphostin, even though neither ovomucoid nor ovalbumin are thought to be direct receptor ligands. The pro-migratory effect of egg is also inhibited by the addition of a transforming growth factor-β (TGF-β)-blocking antibody [25]. Many other peptides with growth factor/cytokine activity have been shown to elicit similar effects in various cell lines, a phenomenon thought to be due to increasing the local production of transforming growth factor β, with TGFβ acting as an intermediary signaler. The susceptibility of growth factor constituents of egg proteins to digestion, during their passage through the gastrointestinal tract, is probably influenced by the presence or absence of other egg or BC proteins, which act as competitive substrates for the digestive enzymes. This is discussed in more detail later, in Section 5 “Effective dosing”.

## 3. Constituents of BC

Although the constituents of BC and mature bovine milk are similar, the relative concentration of macro-nutrients and bioactive molecules is markedly different. The major constituents, including bioactive factors are briefly discussed below. Readers interested in a detailed review of BC constituents are referred to reviews by Playford et al. [27], Poonia et al. [28], and Arslan et al. [29].

### 3.1. Macro-Nutrients and Micro-Nutrients

#### 3.1.1. Proteins and Peptides

BC contains higher total protein, immunoglobulin, and casein content compared with mature milk [27,28]. Whey and casein comprise soluble and insoluble proteins, respectively, with both contributing to the nutritional and bioactivity properties. For example, casein contains peptides with opioid-type and immune modulatory activity [30,31] and, along with the bovine trypsin inhibitor, probably contributes to the preservation of bioactivity and facilitates the adsorption of bioactive peptides/proteins, through reducing digestion from pancreatic proteolytic enzymes [32]. Therefore, in addition to being a source of energy, it may also contribute to antimicrobial, immunological, and anti-inflammatory activity. Whey protein contains multiple bioactive components including IgG, lactoferrin, lactoperoxidase, α-lactalbumin, glycomacropeptide, β-lactoglobulin, and growth factors. It is also relevant that partial hydrolysates of whey and casein may influence innate immunity, acting through toll-like receptors [33]. However, caution in interpretation needs to be shown, as these effects have only been assessed in vitro, using peripheral blood macrophages and the human kidney cell line HEK293. It would, therefore, be of interest to see whether similar results are seen using human gut cell lines and in in vivo models.

#### 3.1.2. Carbohydrates

BC contains many carbohydrates in the forms of lactose, glycoprotein, glycolipid, and nucleotide sugars [27,28,29,34]. Oligosaccharides are present at about twice the concentration of that found in mature milk and, along with glycosylated proteins, may act as prebiotics by being substrates for colonic bacteria [29,34].

#### 3.1.3. Fats and Lipids

The fat content of BC mainly resides within milk fat globules. Amongst these constituents, gangliosides and phospholipids are polar lipids involved in multiple functions such as neuronal development, binding of pathogens, and immune activation [27].

#### 3.1.4. Vitamins and Minerals

BC contains water-soluble and fat-soluble vitamins, usually at higher levels than mature milk, in addition to being rich in calcium, iron, zinc, magnesium, manganese, copper, and phosphorus [29].

### 3.2. Bioactive Components

Bioactive components within BC have the potential to affect multiple physiological pathways including those relating to growth, development, and immunity. Some of the major components are briefly discussed below.

#### 3.2.1. Antimicrobial Factors

BC is rich in immunoglobulins, mainly in the form of IgG [35]. The maternal cow is naturally exposed to a wide range of pathogens and develops specific IgGs against them. These are passed intact into the bloodstream of the suckling calf. It is, therefore, important to note that this is distinct from the adult human situation of ingesting BC, where any immunological effects caused are not due to bovine IgG entering the blood stream intact, as they are probably digested within the gut lumen. Nevertheless, the IgG within BC immunoglobulins may still be involved in preventing microbes binding to host cells, facilitating pathogen presentation to macrophages, stimulating B and T cell activation, and changing gut microflora.

Several other components of BC also possess antimicrobial activity. These include lysozyme, lactoperoxidase, and lactoferrin, which are damaging to both Gram positive and negative bacteria [35]. Lactoferrin has many functions, including increasing iron absorption and may have value for the prevention of necrotizing enterocolitis in premature babies [36,37,38,39]. Several trials have been conducted examining the value of supplementing lactoferrin to enteral feeds. However, a Cochrane systematic review stated that, while there was some evidence of benefits, most trials were of low quality and additional data are required [36].

#### 3.2.2. Cytokines

Cytokine content within BC includes tumor necrosis factor-α, granulocyte-macrophage colony-stimulating factor, and interleukins (IL)-1β, -6, and -10 [40,41]. Growth factors and cytokines are usually considered separately. However, the same molecules may show activity within both categories, such as influencing immunity and stimulating cell growth/repair.

BC contains colostrinin, also known as proline-rich polypeptide, or PRP, is a naturally occurring mixture of proline-rich polypeptides derived from BC. Colostrinin has been shown to help combat excessive inflammatory responses. Evidence in support of this idea includes findings from animal models that colostrinin prevents allergic inflammation due to common and outdoor allergens in a murine allergic airway inflammation model [42]. Similarly, immunocompromised rats infected with enterotoxigenic *E. coli* had reduced endotoxin levels and infected lymph nodes when treated with colostrinin [43].

#### 3.2.3. Growth Factors

In addition to the small molecules that affect growth and are sometimes referred to as preferred substrates, e.g., glutamine, nucleotides, and polyamines, there are the more classical growth factor peptides and proteins that act via receptor binding and cell signaling and influence growth, differentiation, and development [44]. These include insulin-like growth factors (*Somatomedins*) and the EGF receptor ligand family that all bind the EGF (c-erb1) receptor. Members of this family include EGF, transforming growth factor (TGF)α, and betacellulin. BC also contains members of the TGFβ family, which may play a role in repair but paradoxically often reduce proliferation in cell culture assay. BC also contains bovine colostral growth factor, which has a strong sequence homology with platelet-derived growth factor and vascular endothelial growth factor, which, as the name suggests, possesses angiogenic activity and may be relevant for increasing local blood supply in conditions such as gastric ulcer [27,44,45]. Although BC contains multiple growth factors, their pathophysiological relevance will depend on their ability to withstand, at least temporarily, digestion by proteolytic enzymes. They also need to have access to their receptors, which may be restricted to basolateral membranes on non-damaged gut mucosal cells. Furthermore, they would need to be able to be absorbed intact and circulate in the bloodstream to elicit effects on distant organs.

#### 3.2.4. Hormones

Hormones within BC include prolactin, growth hormone, somatostatin, oxytocin, insulin-like growth factor-1, luteinizing hormone-releasing hormone, calcitonin, thyroid-stimulating hormone, thyroxine, progesterone, and estrogen [27]. However, the relevance of peptide hormones within BC, for subjects taking BC orally, is unclear as it is likely that virtually all are digested to inactive forms and do not enter the circulation intact. This idea is supported by the findings that volunteers who ingested 40 g BC as a single dose or 20 g per day for 12 weeks showed no increase in serum insulin-like growth factor-1 levels [46]. 

## 4. Studies of Potential Clinical Benefit

### 4.1. Egg Alone

A major focus on the nutraceutical potential of egg protein relates to the antimicrobial activity of IgY against a wide range of microbes [19]. A potential advantage of using polyclonal IgY, rather than an antibiotic, is in reducing the risk of developing microbial resistance. The immunization of chickens against a specific pathogen results in hyperimmune IgY, which has shown benefits for conditions such as rotavirus infection. A meta-analysis of clinical trials employing IgY against rotavirus identified 2626 subjects from 17 randomized clinical trials. Of these, 1347 subjects received oral IgY and 1279 subjects received conventional treatment, with a statistically significant benefit of the IgY-treated group (odds ratio [OR]  =  3.87, 95% confidence interval [CI] (3.17, 4.74), *p*  < 0 .00001) and (OR  =  3.63, 95% CI [2.75, 4.80], *p*  <  0.00001) [47]. Other potential areas of clinical interest for hyperimmune IgY include dental caries, influenza, and acne, although studies are predominantly at the in vitro and in vivo model stage, with much more work being required to establish any clinical application. For example, Bachtiar et al. evaluated the effect of soybean milk containing IgY against the cariogenic bacterium *Streptococcus mutans* in rats and showed a decrease in the number of *S. mutans* in dental biofilm [48]. Further discussion of hyperimmunized IgY goes beyond this review. However, it is important to note that “natural” non-immunized egg powder also possesses antimicrobial activity against a wide range of pathogens. For example, using human gut monolayer models, egg powder from non-immunized hens was shown to provide protective activity against a broad spectrum of gut-relevant bacteria microbes including *E. coli*, *Klebsiella*, and *Pseudomonas* [49]. It is, however, uncertain how much of this activity was mediated through IgY versus the antimicrobial effects of lysozyme, avidin, beta defensins, and ovotransferrin, along with partially digested peptide fragments exhibiting antimicrobial activity, as discussed earlier.

An additional area where egg proteins may have therapeutic value includes their macro- and micro-nutrient content for maintaining skeletal muscle health and in reducing sarcopenia. The type of protein provided in a meal is critical for sarcopenia associated with aging, with several studies reporting animal proteins, including milk, beef, and eggs, being particularly effective in promoting anabolism in elderly populations. For example, a small clinical study involving 12 elderly women in a crossover protocol showed significantly greater net protein synthesis following a high animal protein diet compared with a high vegetable protein diet [50]. For an excellent review of the area, see Puglisi et al. [51]. 

Egg proteins have also been shown to act as antioxidants in several in vitro and in vivo models; for example, a porcine model of oxidative stress showed that the administration of egg yolk proteins increased GSH and γ-glutamyl cysteine synthetase mRNA expression in the gut wall and circulating red cells [52]. This topic is discussed in detail in Benedé et al. [24]. They conclude that, although several compounds and peptide sequences have shown antioxidant effects using in vitro and in vivo models, the current lack of clinical data means claims of clinical benefit remain speculative.

Other potential applications for egg-derived products include anti-cancer and anti-hypertensive activity, although the current evidence is extremely limited [53]. Several egg proteins and peptides have been reported to induce apoptosis in cancer cells, protect against DNA damage, decrease the invasive ability of cancer cells, and exhibit cytotoxic and anti-mutagenic activity in various cancer cell lines [53]. Some studies have also been extended to in vivo models. For example, the oral administration of hen egg-white lysozyme to mice bearing B16 melanoma significantly reduced the formation of spontaneous lung metastases and prolonged the survival of the treated hosts [54]. These effects were independent of the direct interaction of lysozyme with tumor cells, suggesting an indirect action such as immunopotentiation [54]. Readers interested in further details are referred to Lee and Paik [53].

Egg-derived peptides/proteins have been shown to influence blood pressure in animal models, mainly acting through the renin–angiotensin–aldosterone axis [55]. These include ovotransferrin and protein hydrolysates, especially tripeptide derivatives [12,55]. For example, the oral administration of the egg white-derived anti-hypertensive peptide Ile-Arg-Trp was shown to significantly reduce the blood pressure of spontaneous hypertensive rats via the ACE2/Ang (1-7)/MasR axis, causing enhanced endothelium-dependent vasorelaxation and reduced vascular inflammation [55].

### 4.2. BC Alone

The current evidence for the clinical use of BC was well summarized in a special edition of *Nutrients* and several other recent reviews [27,28,29,56,57,58,59,60] and is, therefore, only discussed briefly. Most bioactive molecules within BC, including IgG, will be digested by acid and digestive enzymes and therefore not absorbed into the bloodstream intact. Disorders of the gastrointestinal tract are, therefore, the area most likely to benefit from oral BC ingestion, as BC components may be able to bind/affect the mucosa directly.

#### 4.2.1. Infectious Diarrhea and Environmental Enteropathy

Several studies have reported positive effects of BC for treating or preventing infectious diarrhea in adults and children. Many of these have been undertaken in low-income countries due to the higher incidence of infectious diarrhea, and suggest that hyperimmunized BC against specific pathogens has value in reducing diarrhea. Positive results have also been reported using non-hyperimmunized BC, especially against rotavirus and *E. coli*-induced diarrhea, due in part to the fact that “non-hyperimmunized” BC contains IgG targeted against many potential pathogens due to natural exposure, including Enterobacter, Klebsiella, and Escherichia coli [61]. It is important to note, however, that in 2019, a meta-analysis on the protective effects of bovine colostrum against childhood infectious diarrhea initially identified 166 research articles, but only 5 randomized controlled trials (RCTs) reached the criteria to be considered in the final analysis [62]. Four of these utilized hyperimmune BC or Ig isolated from hyperimmune BC [63,64,65,66] and one used “standard” BC [67], with the authors concluding that bovine colostrum significantly reduced stool frequency (95% CI: −2.70, −0.14), occurrence of diarrhea (OR = 0.29, 95%CI 0.16, 0.52), and pathogen detection (95%CI 0.08, 0.71) [62]. Since that analysis was undertaken, a double-blind randomized control trial of BC for acute diarrhea in 160 children under 5 years has been conducted in Egypt. The BC-treated group showed a significantly reduced frequency of vomiting and diarrhea and reduced Vesikari (clinical severity) scores after 48 h compared with the control group [68]. Related to these findings, a recent study examined the value of non-hyperimmunized BC for severe acute malnutrition in young children in Zambia and Zimbabwe. This condition is usually associated with underlying gut enteropathy and diarrhea. Results showed that, compared with standard World Health Organisation treatment, it significantly reduced gut and blood markers of inflammation and stimulated gut regeneration [69]. For further details of studies of BC in diarrhea, see Chadwe and Kelly [58].

#### 4.2.2. Non-Steroidal Anti-Inflammatory Drug (NSAID)-Induced Gut Injury

NSAIDs are widely prescribed and effective in the treatment of musculoskeletal injury and chronic arthritic conditions [70]. However, up to 70% of patients with long-term NSAID ingestion have endoscopic abnormalities, such as mucosal erosions, ulceration, and subepithelial hemorrhage, despite only 10% complaining of dyspeptic symptoms [70]. While acid suppressants are effective in reducing gastric injury induced by NSAIDs, they are less beneficial in preventing small intestinal injury. Several researchers have reported the beneficial effects of BC administration using rodent models of NSAID-induced gastric and small intestinal injury [27,71]. A study from Japan using “late colostrum”, which is BC collected post day 5 of calving, suggests that the bioactivity involved in these effects is mainly present in the casein fraction [72]. While the in vivo evidence is relatively strong, there is only limited clinical evidence of the beneficial effects of BC against NSAID-induced gut injury. In a small placebo-controlled randomized crossover study in human volunteers, BC prevented the rise in small intestinal gut permeability, an indirect measure of gut damage, induced by the NSAID indomethacin [73]. Further clinical studies are, therefore, needed to establish therapeutic value.

#### 4.2.3. Inflammatory Bowel Disease (IBD)

Inflammatory bowel disease (IBD) is a chronic relapsing inflammatory disease of the gastrointestinal tract, affecting both children and adults [74]. The two major types are ulcerative colitis, usually restricted to the colon, and Crohn’s disease, mainly affecting the distal small intestine. Current treatment options for IBD include aminosalicylates, immune modulators, and surgery, although treatments are still sub-optimal and new/novel approaches are required [74]. Several studies have reported that BC is effective in animal models of IBD, such as TNBS-induced colitis [75], although animal models have limitations regarding extrapolation to human IBD [76]. There is limited evidence from clinical trials regarding potential efficacy. BC improved the symptoms and histological scores of adult patients with distal colitis who received colostrum enemas in addition to mesalazine compared with controls who only received mesalazine [77]. Indirect evidence is also provided by the finding that TGF-β-supplemented enteral feeds improved clinical outcomes, such as the pediatric Crohn’s disease activity index (PCDAI), in children with Crohn’s disease [58,78]. TGF-β is a major growth factor present in BC, suggesting a potential benefit of BC as an adjunct therapy in patients with IBD.

#### 4.2.4. Sports Medicine and Intestinal Integrity

Athletes undertaking strenuous exercise have an increased incidence of acute gastrointestinal complaints, such as stomach cramps, nausea, and diarrhea [79]. These have potential negative effects on performance and recovery. The underlying causes of these symptoms are incompletely understood, although one area of interest is exercise-induced gut hyperpermeability, caused by mechanisms such as heat and oxidative stress. This increased gut permeability may allow the translocation of luminal toxins and bacteria into the systemic circulation. Pharmacological options to reduce symptoms are limited, especially for elite competitive athletes, increasing the interest in natural-based products for this issue.

Several randomized controlled trials have demonstrated a positive effect of BC on mitigating exercise-induced increased gut permeability, as assessed by changes in dual sugar permeability, intestinal fatty acid binding protein, systemic bacterial DNA, and stool zonulin [80,81,82,83]. As an example, a crossover RCT involved participants running for 20 min on a treadmill after 2 weeks of supplementation with BC or a protein and energy-matched milk protein concentrate placebo. Running caused a 2.5-fold increase in intestinal permeability in the placebo group compared with baseline. BC did not affect baseline gut permeability but truncated this exercise-induced rise by 80% [80]. While these are encouraging results, not all studies have shown a beneficial effect of BC on gut permeability [84,85]. The potential reasons for these varying outcomes include differences in exercise protocols and ambient temperature, along with the source and dose of BC used. Detailed discussion goes beyond the scope of this article and interested readers are referred to Davison [59].

#### 4.2.5. Necrotizing Enterocolitis (NEC)

Unlike the normal adult situation, the premature neonatal human gut may allow luminal bioactive peptides to pass into the mucosa and be absorbed into the bloodstream. There is, therefore, interest in the use of BC for necrotizing enterocolitis (NEC). NEC mainly affects premature infants, with mortality rates of 20–30%. The anti-bacterial, anti-inflammatory, and growth factor content of BC has made it an attractive candidate for the prevention of NEC. While promising results have been found in animal models (especially pig) [86], the limited number of clinical trials regarding the use of BC for the prevention and treatment of NEC has been less optimistic [87]. In 2020, a meta-analysis examined all RCTs comparing colostrum administration vs. placebo or no intervention in preterm infants (gestation age < 34 weeks or birth weight < 1500 g). Overall, nine RCTs (*n* = 689) were included. Unfortunately, no statistical significance in terms of the incidence of NEC (RR = 0.59, 95% CI = 0.33–1.06, *p* = 0.08), late-onset sepsis (RR = 0.78, 95% CI = 0.60–1.03, *p* = 0.08), or mortality rate (RR = 0.63, 95% CI = 0.38–1.05, *p* = 0.07) were found between BC and non-treated groups [88]. For further details of this topic, see Sangild et al. [39], Ghosh and Lacucci [56], and Chadwe and Kell [60].

### 4.3. Egg and BC Combination Therapy

**Figure 1:**
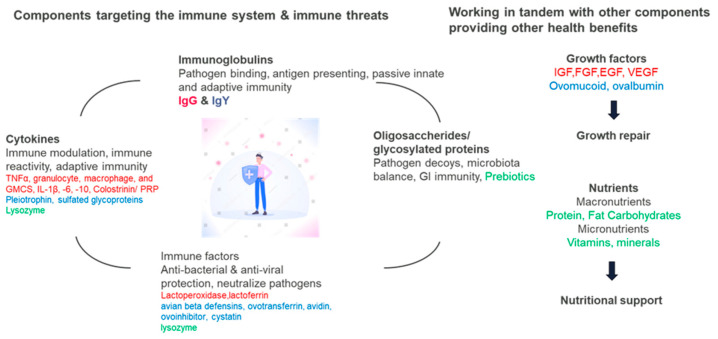
Egg and BC have multiple complementary modes of action. Major pathways in enhancing immunity are shown, with the contribution of specific molecules from BC (red), egg (blue), or same molecule in both (green). Major nutrients and growth factors in egg and BC are also shown.

As discussed above, egg and BC contain multiple antimicrobial, immunomodulatory, and repair components essential for host development/defense functions. Although some bioactive components overlap in both products, e.g., lysozyme, many others do not. There is, therefore, optimism that enhanced activity across a spectrum of conditions may be found if egg and BC are combined, acting through complementary signalers and pathways (see Figure 1).

#### 4.3.1. NSAID and Inflammatory Bowel Disease

**Figure 2:**
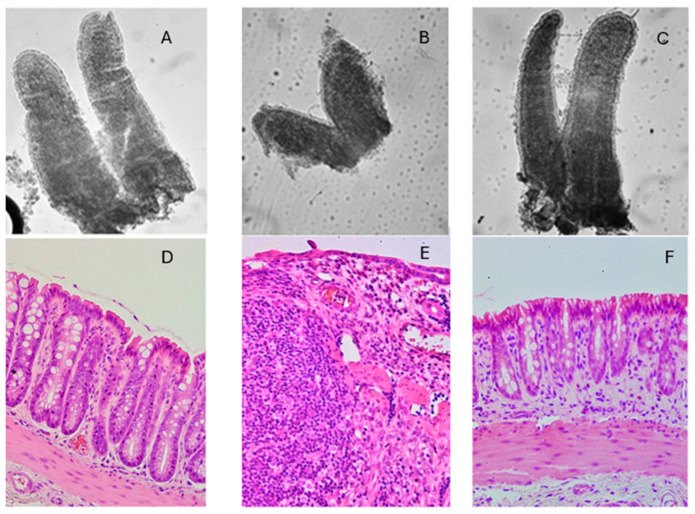
Effect of egg + BC combination therapy on NSAID-induced small intestinal injury in mice and DSS-induced colonic injury in rats. (**A**) Normal small intestinal villi, (**B**) villi from animals treated with NSAID alone, (**C**) villi from animals who received NSAID + egg + BC, (**D**) normal colon, (**E**) colon of animals treated with DSS to cause colitis, (**F**) colon of animals given DSS and egg + BC. Egg + BC resulted in preservation of a nearly normal appearance of villi and colon. Protective effects were significantly higher with egg + BC, rather than egg or BC alone at the same total dose [25]. Images reproduced from Playford et al. [25].

The concept of added value/synergy when using egg and BC together is supported by several in vitro and in vivo studies. Experiments using the gut cell lines AGS, RIE1, and Caco-2 showed synergistic responses in cell proliferation and pro-migratory activity if a 40:60 egg/BC ratio was used, compared with either egg or BC alone at the same total concentration [25]. In the same paper, in vivo studies examined the protective effect of egg alone, BC alone, or in combination in an NSAID-induced small intestinal injury model and in a DSS-induced colitis model. Both models demonstrated that using the combination had greater efficacy in reducing gut damage and inflammation than giving egg or BC alone at the same total concentration (Figure 2) [25]. Clinical studies on the efficacy of combination treatment for NSAID-induced injury or inflammatory bowel disease have not yet been performed and are required to determine whether these results translate to the clinical situation. 

#### 4.3.2. Environmental Enteropathy and Severe Growth Stunting

Combination therapy with egg and BC shows early promise for the treatment of children with severe growth stunting in developing countries. This condition is often associated with environmental enteropathy, where repeated gut infections and inflammation damage the bowel lining. This causes shortening of small intestinal villi, reducing absorptive ability of nutrients, increasing metabolic requirements, and allowing bacterial colonization of the small intestine [89]. A randomized controlled trial of three-months treatment with egg and BC versus control in 277 young children in Malawi showed benefits in reducing linear stunting, reducing gut permeability, and increasing the content of the probiotic *Streptococcus thermophilus* in the stool [90]. Interestingly, there was no substantial difference in the 16S configuration of the fecal microbiota between children receiving egg+BC and control, suggesting the positive effects may not have been mediated through the microbiome. As highlighted by the authors, limitations of the study included a relatively short period of treatment when dealing with linear growth, and the need for caution when extrapolating away from a similar African population. The cost of treatment was about USD 0.20/day, which, while reasonable for Western populations, may be restrictive for government health services and rural subsistence families in Africa.

#### 4.3.3. Small Intestinal Bacterial Overgrowth

Small intestinal bacterial overgrowth (SIBO) is defined as the presence of excess bacteria within the small intestine. SIBO causes bloating, flatulence, diarrhea, and abdominal discomfort. Symptoms of SIBO overlap those of irritable bowel syndrome, although both conditions may coexist. Risk factors for SIBO include altered intestinal anatomy, pancreatic insufficiency, hypothyroidism, and medications that impair gut motility (e.g., opiates) or reduce acid secretion, e.g., proton pump inhibitors [91].

A mixed population of bacteria is often found in patients with SIBO, with some of the commonest species being aerobes such as *Streptococcus*, *Escherichia coli*, *Staphylococcus,* and *Klebsiella* and anaerobes such as *Bacteroides*, *Lactobacillus,* and *Clostridium* [91,92]. Egg and BC combination therapy shows early promise as a new therapeutic approach to prevent or treat SIBO. In vitro studies examining the effect of egg alone, BC alone, and egg + BC have shown that each can stabilize the gut mucosa against a wide variety of microbes, including those commonly associated with SIBO, along with protecting against the very toxic bacterial strains enteropathogenic *Escherichia coli* and Salmonella. Positive effects included reducing bacterial translocation and gut cell apoptosis, in addition to stimulating gut protective factors such as ZO1 and claudin-1 levels. The relative potency of egg, BC, and egg + BC in producing these effects varied, depending on which microbe and which protective factor was measured [49]. These initial in vitro positive results will need to be followed up with efficacy trials on patients with SIBO, using techniques such as breath test assessment, to establish their clinical value.

#### 4.3.4. Glucagon-like Peptide (GLP-1)-Related Gastrointestinal Symptoms

**Figure 3:**
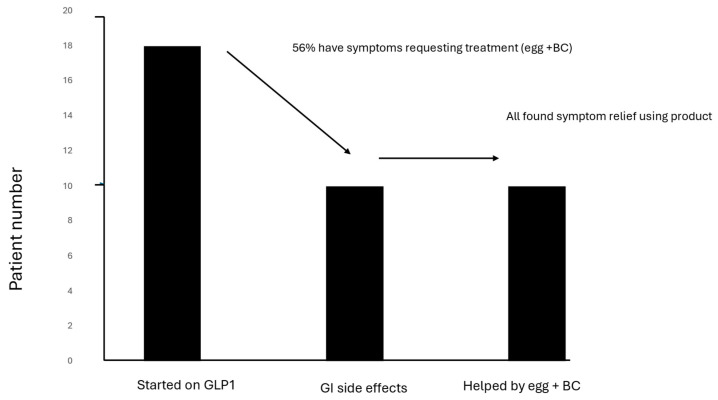
Pilot study of the effect of egg + bovine colostrum (BC) on gastrointestinal (GI) symptoms caused by the initiation of glucagon-like peptide-1 (GLP-1) therapy, based on data provided by [94].

Treatment with GLP-1 therapy for anti-obesity and diabetes control is increasing rapidly. However, its use is hampered by the high incidence of gastrointestinal side effects, such as nausea, vomiting, bloating, diarrhea, and constipation, which occur in 40–70% of patients and may cause patients to discontinue therapy [93]. The current advice to alleviate symptoms mainly relates to reducing high volume meals and other dietary recommendations, with a reluctance to introduce polypharmacy, such as anti-nausea or motility-altering medication. The use of egg and BC is, therefore, appealing as a novel natural-based approach, stabilizing the gut mucosa, providing micro-nutrients at a time of calorie deficit, and addressing dysbiosis, particularly as GLP-1 slowing the gut transit may increase the risk of SIBO development [91]. In support of this idea, a pilot qualitative study of the value of egg and BC to alleviate GI symptoms in 18 patients starting GLP-1 therapy has been performed. They reported that 10 patients suffered gastrointestinal symptoms of sufficient severity that they requested to try egg and BC, with all 10 reporting a symptomatic benefit while taking the product (Figure 3) [94]. These initial positive results are encouraging and provide incentive to perform follow-on studies, such as double-blind placebo-controlled randomized trials to establish clinical value and explore the mechanisms of action.

## 5. Limitations and Caveats on Published Studies of Egg and BC

Limitations in the interpretation of results for the use of egg, BC, or combination treatment can be considered as those generic to nutraceutical research and those specific to the products under examination. These are discussed below.

### 5.1. Regulation of Health Claims

Nutraceuticals are usually marketed as health food supplements, rather than a medicine or medicinal food, both of which require a doctor’s prescription. For regulatory reasons, health food supplements cannot make specific medical claims. Advertising for BC, and to a lesser extent for egg, therefore, usually uses terms such as “to support immune or digestive health”, although there is a wide variation in marketing material regarding claims on what conditions these products may be beneficial for and their relative potency against their competitors.

### 5.2. Small Number of High-Quality Clinical Trials

Compared with most nutraceutical products, there is a strong peer-reviewed publication base using in vitro and in vivo models regarding the potential value of BC for a wide variety of conditions. Similarly, egg, especially as a macro-nutrient and for its IgY antimicrobial activity, has been extensively researched for a variety of clinical situations. However, as highlighted throughout this article, and usually stated at the end of every publication quoted, there is a serious paucity of high-quality clinical trial data. There are several reasons why this is the case; compared with pharmaceutical companies, most of the producers of nutraceuticals are relatively small. The prohibitive costs of major clinical trials means that, compared with trials of medicines from large pharmaceutical companies, nutraceutical clinical studies tend to involve low numbers of participants. Furthermore, as the major market is in long-term health support, clinical trials would require prolonged studies over many years with large numbers of participants, further increasing the costs.

### 5.3. Quality Assessment and Bioactivity

As both egg and BC have a multitude of components, the identity of the active agent(s) is often unclear and may vary depending on the clinical indication. Ensuring consistency in content relevant to its clinical usage is, therefore, much harder than for a single pharmaceutical agent. Maintaining the consistency of the macro- and micro-nutrient contents of egg is relatively easy to do, although these are known to be affected by several factors including the feed given to the hens, the breed of hen, and the temperature of egg storage [95]. There do not appear to be any data, however, regarding how these factors may influence the egg powder’s ability to influence physiological processes such as growth and repair. Studies examining differences in bioactivity using simple in vitro assays of cell proliferation, migration, and immune activation/suppression would provide additional insight into this area.

Similarly, the analysis of total protein and IgG content is usually used as a marker of “quality” by commercial producers of BC. There is some merit in this, as IgG levels, along with many of the growth factors, fall rapidly during the first few days after calving [96]. However, the limitations of only using these measures have been demonstrated in studies examining the bioactivity, assessed by in vitro proliferation and migration assays and an in vivo NSAID-induced gut damaging model, of twenty different commercial BC preparations. Results showed that the bioactivity varied six-fold, despite having similar total protein and IgG content [96]. This may be partly due to differences in the collection time of the BC post calving. However, that is probably not the major reason, as studies on serial samples from a cohort of recently calved animals showed that the bioactivity of BC fell approximately in proportion to protein content during this period, while differences in the bioactivity of commercial samples remained, even when normalized for protein content [96]. Bioactivity is, therefore, probably also being affected by actions occurring post collection, either by the manufacturers or distributors during the processing or storage of product. The bioactivity of BC is highly sensitive to heat exposure [96], and differences in pasteurization techniques may provide a partial explanation. Manufacturers of BC-containing consumer products that undergo a heating/baking stage, therefore, need to be aware of the potential risk of loss of bioactivity, such as pro-reparative growth factor activity or, in the case of IgG, antigen binding. This is despite the concentration of IgG or individual growth-factor levels appearing unchanged. As a result of these findings, several authors suggest that bioassays demonstrating effects such as IgG antigen binding and/or proliferative activity should be performed before embarking on clinical trials. These would provide more clinically relevant data than quantitating potentially inactivated peptides and proteins by measuring total IgG or a particular growth factor content using an immunoassay. Claims of enhanced immune or growth bioactivity of any marketed product should, therefore, be based on the results of a relevant bioassay rather than relying on IgG, total protein, or individual growth factor quantitative assays [96].

### 5.4. Effective Dose

Because there are very few studies available that examine dose responses in clinical trials, the optimal dose of egg, BC, or combination for human use is uncertain. This issue is further confounded by the fact that different formulations of egg, BC, or combination will result in different stabilities of growth factors and immune factors, such as IgG, during their transit through the stomach and small intestine. When taken by mouth, egg, BC, or combination are exposed to hydrochloric acid and pepsin in the stomach, followed by bile and pancreatic enzymes, such as trypsin and chymotrypsin, in the small intestine. Studies have shown that the growth factor bioactivity and immunoglobulin binding ability of egg or BC, assessed using in vitro and DSS-induced colitis models, is adversely affected by HCl/pepsin/trypsin/chymotrypsin exposure, resulting in a fall of bioactivity of up to 60%. However, this can be partially mitigated by the presence of specific proteins such as casein, soya bean trypsin inhibitor, or egg ovomucoid, which act as preferred substrates for the proteolytic enzymes [26,97]. The presence of these specific proteins is therefore likely to cause at least temporary stabilization of the growth factor and immune components within the gut lumen, allowing them to travel intact to distal gastrointestinal parts to enhance gut integrity and repair [26,32,97]. Interestingly, not all proteins have this protective effect, with lactalbumin, which is present in the whey fraction of BC, not showing any protective ability [32]. This phenomenon is probably related to the relative affinity of pepsin/pancreatic enzymes to the protein versus the growth or immune factor. These in vitro and in vivo results suggest that combining egg and whole BC has the added potential advantage of maximizing the biostability of immune and growth factors, through egg protein constituents such as ovomucoid and the casein content within whole, defatted, or minimally processed BC. The direct clinical observation of these potential protective effects within the stomach and small intestine would be of interest but would be logistically difficult to perform. This has been partially achieved by examining the stability of EGF when exposed to human fasting small intestinal juice aspirate, where similar results of preservation of the bioactivity of EGF by casein and soya bean trypsin inhibitors were obtained [32]. It is, therefore, important that the formulation of egg, BC, or combination being administered is taken into consideration when determining the probable relative potency and dosing requirements for human use.

### 5.5. Safety

Egg and milk-containing products are part of a staple diet of most of the Western population. Although both egg and BC are generally considered safe, consumers with egg or milk allergies or intolerance should probably avoid their use. For subjects with lactose intolerance, some BC products are available lactose-free. More caution and monitoring would need to be shown if high dose individual proteins or peptides derived from egg or BC are being tested or used in a clinical setting.

## 6. Conclusions

Egg and BC are rich sources of macro- and micro-nutrients and bioactive molecules that have potential relevance for immunity, growth, and repair. In the health food supplement market, egg and BC are considered as comprehensive “superfoods”. Acceptability by the public is also enhanced by their safety profile and links to nature. Compared with most health food supplements, there is a relatively strong in vitro and in vivo peer-reviewed publication record, suggesting potential benefits for a wide variety of indications, including gastrointestinal conditions. Synergistic responses have been demonstrated for the combination of egg and BC using in vitro and in vivo assays but need to be supported by clinical data. Evidence-based systematic reviews of the efficacy of egg and BC are often hampered by heterogenous and poor-quality studies. More translational studies are required to establish their value in clinical practice.

## Abbreviations

BC—bovine colostrum, EGF—epidermal growth factor, EGFR—epidermal growth factor receptor, GLP-1—glucagon-like peptide -1, IBD—inflammatory bowel disease, IGF—insulin-like growth factor, IL—interleukin, NEC—necrotizing enterocolitis, NSAID—non-steroidal anti-inflammatory drug, PRP—proline-rich polypeptide, RCT—randomized clinical trial, SIBO—small intestinal bacterial overgrowth, TGF-—transforming growth factor-, URTI—upper respiratory tract infections, WHO—World Health Organisation.
